# Application of Proteomics Analysis and Animal Models in Optic Nerve Injury Diseases

**DOI:** 10.3390/brainsci13030404

**Published:** 2023-02-26

**Authors:** Zhaoyang Meng, Ran You, Arif Mahmood, Fancheng Yan, Yanling Wang

**Affiliations:** 1 Department of Ophthalmology, Beijing Friendship Hospital, Capital Medical University, Beijing 100050, China; 2 Center for Medical Genetics and Hunan Key Laboratory of Medical Genetics, School of Life Sciences, Central South University, Changsha 410078, China

**Keywords:** optic nerve injury, proteomics, bioinformatics, retinal ganglion cells, ophthalmopathy

## Abstract

Optic nerve damage is a common cause of blindness. Optic nerve injury is often accompanied by fundus vascular disease, retinal ganglion cell apoptosis, and changes in retinal thickness. These changes can cause alterations in protein expression within neurons in the retina. Proteomics analysis offers conclusive evidence to decode a biological system. Furthermore, animal models of optic nerve injury made it possible to gain insight into pathological mechanisms, therapeutic targets, and effective treatment of such injuries. Proteomics takes the proteome as the research object and studies protein changes in cells and tissues. At present, a variety of proteomic analysis methods have been widely used in the research of optic nerve injury diseases. This review summarizes the application of proteomic research in optic nerve injury diseases and animal models of optic nerve injury. Additionally, differentially expressed proteins are summarized and analyzed. Various optic nerve injuries, including those associated with different etiologies, are discussed along with their potential therapeutic targets and future directions.

## 1. Introduction

The optic nerve (ON) is an important sensory nerve made of axons composed of myelinated nerve fibers. This nerve connects to the brain and the retina to transmit vision. Five different types of neurons run through the retina to connect to the thalamus [1]. Retinal ganglion cells (RGCs) are central neurons located in the innermost layer of the retina and are the main neurons that constitute the retinal nerve fiber layer [2]. When photoreceptors receive visual signals, they transmit the visual information to interneurons, and then the interneurons process the information to transmit it to the RGCs. Horizontal cells, bipolar cells, and amacrine cells are the main neurons involved in photoreceptor signals [3,4]. When the photoreceptors receive a visual signal, they transmit the visual information to the interneurons, and the interneurons process the visual information to transmit it to the RGC [5], then pass it through the unmyelinated axons to the lateral side of the contralateral geniculate body of the brain and connect with the neurons of the superior colliculus. The dura mater in the brain acts as a myelin sheath and covers a large area on the surface of the optic nerve [6].

The optic nerve serves as an important bridge between the eyes and the brain. Blood supply to the optic nerve primarily comes from the distal branches of the intraretinal ophthalmic artery and ophthalmic vein [7]. It is also the first branch of the internal carotid artery after passing through the cavernous sinus [8]. The central retinal artery contains numerous arterioles to provide blood supply to the entire optic nerve, and numerous optic nerves form a blood supply network by capillaries to maintain the connection between the retina and the sclera. When retinal blood vessels are blocked, the blood supply to the optic nerve is reduced, and consequently, optic nerve damage occurs.

Proteomics is a powerful platform for exploring single proteins and complex protein samples. Combining the techniques of gel- and chromatography-based separation enables processing of questions from all areas of basic science and medicine. Proteomics has been widely used to study eye diseases, and numerous studies have collectively identified over 40 proteins in different optic nerve injury-related diseases [9,10,11,12,13]. Proteomics analysis on different optic nerve damage diseases in humans and animal models (including glaucoma, diabetic retinopathy, retinal arteriovenous occlusion, optic nerve transection model, retinal ischemia-reperfusion model, intraocular hypertension model, optic nerve crush injury model, and traumatic optic neuropathy) have revealed possible diagnostic proteins and their mechanistic pathways [14] in addition to potential therapeutic protein molecules and their mechanisms [15,16,17]. 

Use of animal models in biological studies are essential to understand specific diseases’ etiologies and pathological mechanisms and the underlying causes of the diseases. Such models provide an effective therapeutic target for drug development. The molecular and cellular mechanisms of optic nerve injury have been studied using a variety of animal models. Optic nerve crush (ONC), axotomy, and ocular blast are the three most common models for studying optic nerve trauma [18]. An optic nerve crush (ONC) model involves surgically exposing the optic nerve and clamping it with forceps or a hemostat for a period of time [5,19,20].

Due to the extremely complex protein composition of the retina, proteomic methods have become the preferred method for analyzing protein changes within it. Compared with the enzyme-linked immunosorbent assay and Western blotting methods, proteomics can obtain more valuable information using simpler experimental operations. Proteins are indispensable sensors and regulators of organisms that directly participate in and regulate the occurrence and development of diseases. Thus, proteomics offers great advantages in the study of disease mechanisms. The combination of proteomic and bioinformatic methods can offer further utility in the discovery of protein changes in various diseases and can also be used to analyze the action pathways of proteins. Proteomics has become an indispensable analysis method in the field of optic nerve eye disease and has helped advance the development of eye disease research. In this review, we summarize and review (i) the application of proteomics in the context of optic nerve injury diseases, (ii) the pathogenesis of optic nerve injury diseases, and (iii) the key proteins involved; we then make recommendations for the future application of proteomics in optic nerve injury diseases.

## 2. Introduction of Optic Nerve Injury Ophthalmopathy and Animal Models of Optic Nerve Injury

Optic nerve damage ophthalmopathy is a common blinding eye disease in clinical practice, and the accompanying optic nerve damage is irreversible. Approximately 2.2 billion people around the globe are suffering vision impairment/injuries [21]. The prevalence and incidence of eye-related disorders and injury vary from region to region. For example, in the US, 24 million people suffered from vision loss/impairment, and 147,000 people are totally blind, while 1.7 million are partially blind by any means [22]. In Western countries, the prevalence rate of only ocular trauma was 14.4 percent to 21.1 percent [23], while in Singapore and China, the prevalence was 4.4 and 3.6%, respectively [24,25]. In addition, the prevalence/incidence of such diseases and other related diseases still either lack an exact figure of prevalence/incidence or are not yet defined well [26]. The optic nerve is composed of numerous retinal ganglion cells and axons, which form an important structure that transmits visual information from the eye to the brain [27,28]. Optic nerve injury disease refers to the visual dysfunction caused by inflammation [5], ischemia, hypoxia, elevated intraocular pressure, and other changes in the optic nerve affecting the visual conduction pathway [29,30]. The optic nerve belongs to the central nervous system [28]. When the optic nerve is damaged, a substantial number of RGCs die, thereby causing changes in the retinal microenvironment and subsequently aggravating optic nerve damage [31]. Optic nerve damage is involved in various eye diseases, such as glaucoma, cataracts, and diabetic retinopathy. Physical trauma, genetics, and other factors can lead to damage to the optic nerve, thereby affecting vision and potentially causing blindness [32,33].

### 2.1. Research Progress in Optic Nerve Injury Ophthalmopathy

#### 2.1.1. Types of Nontraumatic Optic Nerve Injury

The optic nerve injury included nontraumatic optic nerve injury and traumatic optic nerve injury. Nontraumatic diseases include glaucoma, diabetic retinopathy, retinopathy caused by hypertension, optic neuroblastoma, and thyroid-induced retinopathy, and these are common in clinical practice [34,35]. Glaucoma is characterized by the progressive death of retinal ganglion cells and axonal loss [36]. Even more serious is the central gaze [31,37]. Glaucoma is divided into primary glaucoma and secondary glaucoma [38]. Primary glaucoma refers to the increase in intraocular pressure and the degeneration of the optic nerve under physiological conditions. Secondary glaucoma is caused by inflammation, trauma, ocular vascular damage, or increased intraocular pressure due to neovascularization, among other causes [39,40]. With the rapid development of medical diagnostics, the early screening of glaucoma relies on the measurement of retinal nerve fiber layer thickness by optical tomography (OCT) combined with intraocular pressure detection. Numerous studies have shown that the use of optical tomography scanners to detect the thickness of the retinal fiber layer can only be effective when the onset of glaucoma reaches a certain point [41,42,43,44,45] such that the retinal nerve fiber layer has been damaged. 

Diabetic retinopathy is a retinal vascular complication. The development of diabetes is related to blood pressure, blood sugar, dyslipidemia, and kidney disease [46,47]. Diabetic retinopathy is primarily a disease of the fundus microvessels. Retinal blood vessels are important transport tissues that provide nutrients and energy support for cells in the retina and the optic nerve. Diseased retinal blood vessels may affect all cells in the retina, thereby resulting in irreversible damage to the optic nerve [48]. Due to the complex pathogenesis of diabetic retinopathy, systemic therapy is the mainstay of treatment and includes the control of blood sugar, blood lipids, and blood pressure, as well as regular fundus examinations [49,50,51,52]. Retinal laser therapy and intraocular anti-VEGF drugs, among other treatments, are used to inhibit neovascularization in the retina. When the intravitreal hemorrhage is excessive such that it cannot be absorbed, vitrectomy is then required [53].

As a result of ischemic optic nerve injury, retinal microvascular occlusion puts the optic nerve in a state of ischemia and hypoxia for a long time, and the optic nerves atrophy. Ischemic optic nerve injury is divided into optic nerve head injury and posterior optic nerve injury in accordance with the site of injury [54,55,56]. Acute optic nerve injury is more common in middle-aged and elderly people over the age of 50 [55]. The site of optic nerve injury is generally within the anterior part of the optic nerve. In these instances, the visual acuity of the unilateral eye typically decreases significantly without pain [57]. There are some cases of bilateral acute optic nerve anterior injury in clinical practice. Posterior optic nerve injury is often caused by ischemia and hypoxia caused by the occlusion of the short posterior ciliary artery, and local optic nerve edema further aggravates the injury.

#### 2.1.2. Diagnosis of Nontraumatic Optic Nerve Injury

Imaging of the retinal fundus is an important tool for the clinical diagnosis of optic nerve damage. The fundus can show the specific state of the blood vessels in the retina of the patient, including the diameter of the blood vessels, the thickness of the wall, the degree of the curvature of the blood vessels, and the existence of new blood vessels. Fundus fluorescein angiography is an invasive diagnostic method for detecting fundus capillary circulation and vascular lesions, which can accurately guide subsequent laser treatment for the fundus lesions [58,59]. Optical tomography is a fast and noninvasive retinal imaging technology that has been widely employed in the detection of retinal layers. Ocular ultrasonography is mainly used for the examination of cataracts or vitreous opacity. As a disadvantage, it cannot identify the blood vessels of the fundus. However, it can detect conditions such as vitreous hemorrhages, proliferative stretched retinas, and retinal detachments accurately [60]. The above examinations can be detected only after the optic nerve is damaged. There is a lack of specific detection points by which to achieve early detection, make an early diagnosis of the disease, and implement early treatment.

#### 2.1.3. Treatment of Nontraumatic Optic Nerve Injury

At present, no effective method exists for the treatment of optic nerve damage caused by optic nerve injury ophthalmopathy. The principal treatment is instead to control the pathogenic factors of optic nerve injury and relieve the clinical symptoms. The optic nerve is difficult to repair and regenerate after damage. Traditional treatment drugs used for optic nerve injury are glucocorticoids, vitamins, and neurotrophic factors, among others, but these drugs can only maintain the condition of optic nerve injury by preventing further deterioration [61,62]. In recent years, new drugs have been discovered that provide resistance to oxidative stress, provide exogenous cytokines, inhibit, or stimulate inflammatory responses, have antiglial scarring effects, cause directional induction of axonal regeneration, or have neuroprotective functions; these include N-methyl-D-aspartate receptor antagonists, Rho agonase inhibitors, and antioxidants, as is well explained by Vishwaraj and colleagues [63]. However, these drugs have not yet been used clinically. Some novel drugs have begun clinical trials, so their long-term therapeutic effects are not yet known. Consequently, there are currently no targeted drugs for optic nerve injury diseases.

### 2.2. Establishment of an Animal Model of Optic Nerve Injury

Establishing a simple, efficient, easy-to-operate, and highly reproducible optic nerve injury model is the basis for the in-depth study of optic nerve injury diseases. An ideal optic nerve injury model should have the following characteristics: (i) the mechanism of optic nerve injury resembles human optic nerve injury, and the process and results of optic nerve injury are as similar as possible to the appearance of human optic nerve injury; (ii) the animals are economical and easy to obtain; and (iii) the model-making process is relatively simple, and the repetition rate is high. The main modeling methods of optic nerve injury include traction injury, crush injury, impact injury, optic nerve transection, clamping injury, compressive injury, toxic injury, and electrical stimulation injury [5,64,65]. Models have been established for clamping injuries and compression injuries. Commonly used animals for establishing optic nerve injury models include rats, rabbits, and mice, and in experimental studies, researchers often choose rats as the first choice. 

Common disease models include glaucoma, cataracts, diabetic retinopathy, traumatic optic nerve injury, and retinal ischemic optic nerve damage ophthalmopathy models. This section provides a brief summary of these optic nerve injury disease models.

#### 2.2.1. Glaucoma Model

**Figure 1:**
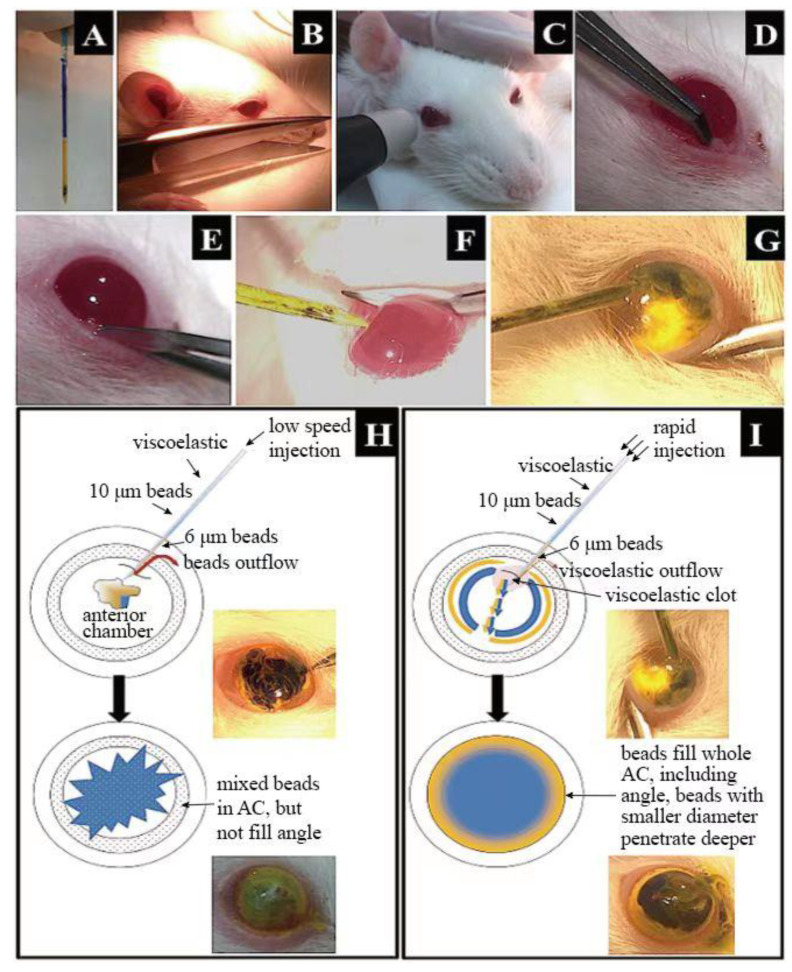
**Glaucoma Model in Wistar rats.** (**A**) Glass needle filled with a suspension of viscoelastic solution, 10 μm beads (blue) and 6 μm beads (yellow). Before the model, (**B**,**C**) preparation occurred, and IOP was measured. (**D**,**E**) Immobilization of the eyeball was performed with microforceps. (**F**) Introducing the glass needle into the anterior chamber at an approximately 45-degree angle to the corneal surface. (**G**) Injection of beads, slight corneal edema visible. (**H**,**I**) Schematic differences between low- and high-speed bead injection. (**H**) With low-speed injection, the beads formed a clot in the center of the anterior chamber, impairing the distribution into the iridocorneal angle. The final outcome is no separation of the beads. (**I**) With rapid injection, high pressure, which is generated in the AC, pushes the beads deeply into the iridocorneal angle. The limited volume of the AC and the high pressure improve the separation of beads and their distribution, which results in higher values of IOP.

As shown in Figure 1, rats are the preferred model animals used for establishing glaucoma models. They are readily available, and their ocular physiology and function resemble that of the human eye. Glaucoma models can be divided into two categories: the experimental glaucoma model and the hereditary glaucoma model [66]. Commonly used models of glaucoma include episcleral venous saline injection, laser photocoagulation, and microsphere injection, all of which are caused by external interventions to increase intraocular pressure.

(1) Episcleral vein injection: a commonly used method for establishing glaucoma models for scientific research purposes is by injecting hypertonic saline into the scleral surface blood vessels of adult mice to establish an intraocular hypertension model [67]. The advantages of this method are that the test materials are easy to obtain, the test method is simple, and the method can be used to model high intraocular pressure to evaluate the effect of ocular hypotensive drugs [68].
(2) Superficial scleral vein-burning method: as above, this modeling method is also a way of increasing intraocular pressure, and the change of intraocular pressure depends on the number of cauterized episcleral veins on the sclera. When the number of cauterized veins equals 4, the intraocular pressure can be increased to 60 mmHg, which can be maintained for several weeks to induce the death of RGCs. Compared with episcleral intravenous injections to establish an intraocular hypertension model (as above), its operation is simpler and more reproducible, the intraocular pressure can be maintained for a stable time, and it can induce the degeneration and apoptosis of RGCs in a way that more closely resembles human chronic glaucoma [69,70].
(3) Microsphere injection: the microsphere injection method uses 10 μm sized latex microspheres to inject into the anterior chamber to prevent the outflow of the aqueous humor and preserve the complete anatomical structure. This method can reduce the inflammatory response. The period whereby high intraocular pressure is maintained is different depending on the size and material of the microspheres. Microspheres of 5–15 μm can raise the intraocular pressure by 5–10 mmHg for 2 weeks [71]. The microsphere method is simple and easy to operate, and the reproducibility is high. At present, the microsphere ocular hypertension model has been widely used in experimental research pertaining to the evaluation of glaucoma drug treatments.

#### 2.2.2. Traumatic Optic Nerve Injury

Traumatic optic neuropathy is an optic nerve damage disease caused by external trauma to the head, face, and orbit [72]. It is divided into direct optic nerve injury and indirect optic nerve injury according to the injury mechanism involved [73,74]. There is currently no good treatment for traumatic optic nerve injury. Appropriate animal models remain an important means of conducting research.

(1) Direct optic nerve injury models

Optic nerve transection (ONT) is a direct optic nerve injury model, and its modeling method is based on directly cutting the optic nerve of the model animal. Advantages of this model include simple manipulation, consistent animal injury, and easy reproducibility [75]. After the successful establishment of the model, the apoptosis of RGCs in the retina increases, the apoptosis of RGCs can be quantified, and both the time until and process of RGC death can be traced while quantifying the death of RGCs [76]. At present, this model has been widely used to study the mechanism of RGC degeneration and apoptosis. This model is mostly used for the evaluation of drug treatments following nerve injury, and it can be used to observe the changes in RGCs to detect the effect of experimental treatments on fundus cells [77]. Because the optic nerve is connected to the nerves in the brain, the model can also be used to study whether the optic nerve is completely cut off from other nervous systems in the brain. This model must be established by surgery, and if the surgical incision is too large, it may cause infection or inflammation in the animal.

**Figure 2:**
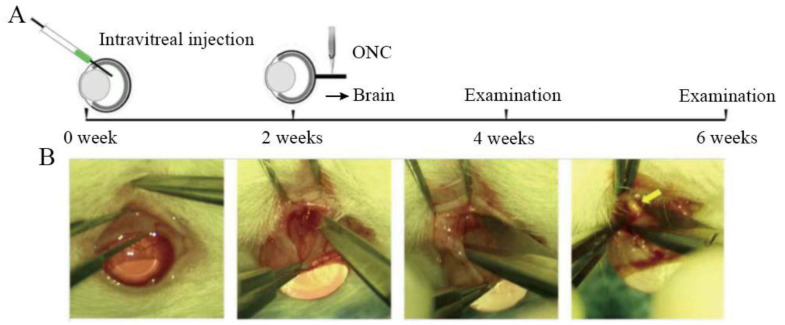
**Procedures of ONC model.** (**A**) Schematic timeline of the experimental procedure. Two weeks after intravitreal injection of adeno-associated virus vectors, ONC was performed on the injected eyes. Analysis, including morphological, functional, and electrophysiological changes of RGCs/ONs, was performed prior to ONC, and 2 and 4 weeks after ONC. (**B**) ONC procedure (magnification, ×6). The yellow arrow indicates the site of the ONC. ONC, optic nerve crush; RGC, retinal ganglion cell.

The optic nerve crush model (ONC) is an important model for traumatic optic nerve injury and is mainly used to conduct research on the regeneration of RGCs and axonal regeneration. The concrete process is manifested as Figure 2. During modeling, attention must be paid to the time and strength of the squeezing pressure applied to the optic nerve to avoid damage to the ophthalmic artery, which can lead to retinal ischemia. Analysis, including morphological, functional, and electrophysiological changes of RGCs/ONs, was performed prior to ONC and 2 and 4 weeks after ONC. At present, the optic nerve compression time used in the ONC model is largely different from that used in other models, thereby resulting in a large difference in the degree of damage caused by the model [78]. The ONC model is widely used to study the death mechanism of optic neurons and in experimental drug research. Compared with the ONT model, the ONC model has the advantages of a smaller opening, clear damage, and a higher survival rate.

(2) Indirect optic nerve injury models

Chronic compressive injury to the optic nerve is used to simulate damage caused by either post-traumatic hematoma or tissue inflammatory edema caused by trauma. The method for establishing this model is simple. With the extension of the time after modeling, the optic nerve damage gradually increases. It is often used to simulate pathological changes such as optic nerve axon ectopic and degeneration caused by the gradual increase in post-traumatic hemorrhage and edema. Disadvantages are that this method is relatively more prone to causing inflammation and infection [79].

The eye shock injury model is used to simulate the type of damage to the optic nerve caused by shock wave explosions. Compared with other optic nerve injury models, this model requires a specific blasting and protection system to simulate explosive injuries. The eyes and head are damaged by the shock wave while the body is protected. The disadvantages of this model are that the mortality rate of model animals is high and the retinal tissue is severely damaged during modeling, which greatly interferes with the detection of retinal tissue after the model has been established.

#### 2.2.3. Ischemia/Ischemia-Reperfusion Optic Nerve Injury

Retinal ischemic ophthalmopathy is primarily caused by central retinal arteriovenous vascular occlusion and retinal ischemic ophthalmopathy induced by vascular occlusion (the latter of which is caused by glaucoma and diabetic retinopathy) [80]. The clinical manifestations are retinal ischemia (which induces optic nerve damage) and further vision loss in patients, which can be caused by the aggravation of retinal damage after drug recanalization. This model is highly useful for conducting clinical research on the mechanism and prevention of retinal ischemic diseases.

Retinal vessel ligation is also a commonly used model of retinal ischemia/reperfusion. In this model, either the internal retinal vein or artery is ligated and then loosened to establish a reperfusion model [81]. Some researchers have also established optic nerve ischemia-reperfusion models by ligating unilateral or bilateral common carotid arteries [81]. It is difficult to establish a simple retinal ischemia model, and hence, most of the established models are retinal ischemia-reperfusion injury models. Compared with other models, the retinal ischemic eye disease model has poor repeatability due to difficulties in maintaining the same degree of damage caused by different surgical methods, different vessel clipping positions, different clipping times, and different times of reperfusion, although two advantages are that the model rate is high and mortality is low. Retinal ischemic diseases are mainly used to study changes in RGCs in the retina, to track the changes in the optic nerve, and to explore the mechanism of optic nerve damage.

## 3. Application of Proteomics in Optic Nerve Injury Diseases

**Figure 3:**
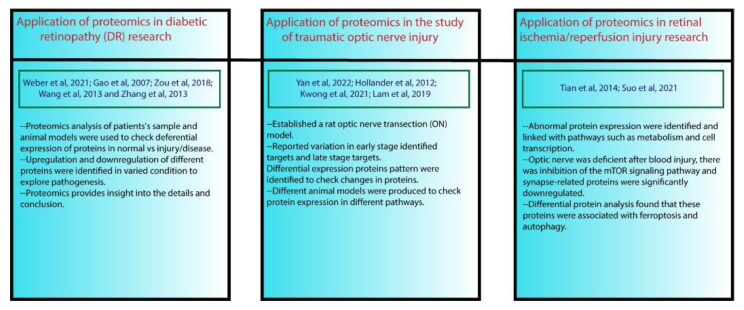
Role of proteomic studies in Optic nerve injuries and its outcomes [82,83,84,85,86,87,88,89,90,91,92].

Optic nerve damage can significantly reduce the vision of patients, thereby having a serious impact on their daily lives and their families. In clinical practice, optic nerve injury is mainly diagnosed by OCT detection and by fundus angiography. However, these two methods are not ideal for detecting early-stage optic nerve injuries. Additionally, there currently exists no effective treatment used in clinical practice for optic nerve damage eye disease. To remedy these two issues, proteomics can be used to analyze the composition and content of proteins in retinal tissue to obtain the changes in proteins during the development of diseases, thus offering the potential to obtain potential diagnostic targets to provide a means of early detection as well as key therapeutic sites to help in the development of novel effective treatments. The outcomes of proteomics analysis are summarized in Figure 3 and well explained below.

### 3.1. Application of Proteomics in Diabetic Retinopathy (DR) Research

Proteomics has been widely used in the research of optic nerve injury diseases. The number of differentially expressed proteins and their main roles were obtained from the literature for this review. Targeted proteomic studies using only ELISA or other immunological assays were excluded. Weber et al. [82] used 23 vitreous samples of proliferative diabetic retinopathy (PDR) and divided them into three groups according to the severity of the disease for tandem mass spectrometry calibration analysis. They quantified 2400 proteins and three PDR differences. Bioinformatic analysis of the proteins found that the glycolysis and gluconeogenesis pathways were activated in the vitreous of PDR patients, while the expression of carbon metabolism-related molecules was upregulated. Gao [83] conducted proteomic analysis on the PDR vitreous of patients with diabetic retinopathy. Compared with the normal control group, there were 119 differentially expressed proteins in the PDR of patients with diabetic retinopathy. Among them, the level of CA-I was significantly increased. Further research found that this protein could increase the permeability of the retina and choroid. Increases in VEGF are also known to promote the formation of new blood vessels in the retina, thereby indicating it to be an important protein for aggravating the disease. Ranibizumab is often used in the treatment of DR in clinics. Zou [84] and others performed proteomic detection on the vitreous humor of eight DR patients, nine DR patients treated with leizumab, and nine nondiabetes patients. Compared with the control group, a total of 72 differential proteins were screened out; compared with the PDR group, there are 3 upregulated proteins and 16 downregulated proteins in the treatment group, which may be one of the important proteins to promote the improvement of PDR. After biological analysis, it was found that these proteins were related to pathways involving “the innate immune response”, “complement activation”, and “proteolysis”. The early detection of PDR is a challenge that has not yet been overcome in clinical work, and this is due to there being a lack of a simple, rapid, and specific molecular markers to detect the progression of PDR. In the plasma of PDR patients, a total of 23 upregulated and 13 downregulated proteins were found, with FCGR3A, DPEP2, and ADGRF5 identified as potential protein markers in the plasma [93]. Wang et al. [85] used RP-HPLC and ESI-MS/MS to detect 96 differentially expressed proteins, 37 upregulated proteins, and 59 downregulated proteins in the vitreous of PDR patients. The key identified differentially expressed proteins were angiopoietin-related protein 6, apolipoprotein AI, estrogen receptor alpha, and tubulin. Zhang et al. [86] used DB mice to build a model of diabetic retinopathy, treated the diabetic retina with phlorizin, and then analyzed the retinal proteomics of the mice in each group by LC-MS/MS to attempt to find effective treatments. The results of the study found a total of 1636 proteins in the mouse retina; 348 differentially expressed proteins were detected in the DR group compared with the blank control group, while only 60 differentially expressed proteins were detected in the treatment group compared with the DR group (including 27 upregulated proteins and 33 downregulated proteins). Bioinformatic analysis conducted on these differentially expressed proteins found that most were involved in processes pertaining to oxidative stress, apoptosis, and energy metabolism, among other pathways. The authors suggested that the ability of phloridzin to inhibit retinal cell apoptosis and slow down the development of DR was associated with the upregulation of γ-crystallin and Glrx-3 in DR mice. The authors believed that the pathogenesis and development of DR are related to the downregulation of Glrx-3 and that γ-crystallin may be an important therapeutic site for improving the clinical symptoms of DR. Winiarczyk [94] used two-dimensional electrophoresis to analyze the tear film of 15 diabetic dogs and 13 normal dogs by proteomic analysis to identify one upregulated protein and eight downregulated proteins in the diabetic group compared to the controls. The authors noted that the only upregulated protein in DR dogs (SRCIN1) is an important mediator of the VEGF pathway, and the observed increased expression of SRCIN1 functions by activating the VEGF/SRC kinase signaling axis, thereby promoting retinal barrier dissolution and retinal neovascularization. Prelamin-A/C, Flotillin-1, Pro-MCH, PI4KIIα, PARP12, PARP/GRIP, TPR36, and Serpin B3 were found to be downregulated in DR dogs, all of which are involved in cellular immunity, inflammation, apoptosis-related pathways, and the Src kinase pathway. In order to observe the changes in lens-related proteins that occur in diabetic retinopathy, Nagai et al. [95] used a STZ-induced diabetic retinopathy model in rats to identify a total of 229 proteins in the lens of the normal control group and 235 proteins in the lens of diabetic retinopathy rats, which included a total of 52 differentially expressed proteins. Among these 52 differentially expressed proteins, superoxide dismutase was significantly downregulated, while phosphorylated p38 was significantly increased. The antioxidant capacity of the eyes of patients with diabetes is known to be weakened, and this is because elevated blood sugar increases the load of ocular oxidants and oxidative free radicals in the eyes. This occurrence changes the overall sensitivity of the lens such that the aggravation caused by glycosylation and the inactivation of antioxidant enzymes can exacerbate the lens opacity velocity. The downregulation of superoxide dismutase is an important factor for activating the P38 pathway, and inhibiting the activation of the P38 signaling pathway may therefore be an important target for the treatment of diabetic cataracts.

### 3.2. Application of Proteomics in the Study of Traumatic Optic Nerve Injury

Traumatic optic nerve injury is one of the more common forms of optic nerve injury. It is characterized by either direct or indirect damage to the optic nerve that occurs when the face or head is damaged by an external force and involves the degeneration or death of retinal ganglion cells (RGCs). Yan [87] established a rat optic nerve transection (ON) model and used iTRAQ proteomics to analyze the retina to detect a total of 4717 proteins. Compared with the blank control group, 708 differentially expressed genes were detected in the ON group. In this study, differentially expressed proteins between different time points were also compared. The findings indicated that potential diagnostic targets in the early stage of the disease were different from those in the middle and late stages of the disease. Pathway analysis of these differentially expressed genes found that these were related to “carbon metabolism” and “the ribosome”. To examine the effect of therapeutic drugs on rats after optic nerve transection, Hollander et al. [88] injected Hepatoma-derived growth factor (HDGF) into the eyes of rats following ON and observed postoperative changes in retinal proteins at 7, 14, and 21 days. A total of 52 upregulated proteins and 19 downregulated proteins were found in the retina of the ON group. Compared with the treatment group, HDGF was able to activate both the MAP and PI3 kinases while also activating the AKT phosphorylation pathway.

To study the changes in RGCs after optic nerve transection in rats, Kwong et al. [89] established a rat unilateral ON model, obtained the retinas of rats 2 weeks after the operation, and analyzed them by quadrupole time-of-flight mass spectrometry (QTOF-MS). Murine retinas were evaluated for their protein contents. A total of 3641 proteins were found, of which 25 were downregulated and 37 were upregulated in the time quadrant, while 5 downregulated proteins and 20 upregulated proteins were found in the nasal quadrant. Differentially expressed proteins differed between different times of injury, which was consistent with previous findings. Among them, CLU, GFAP, GNG5, IRF2BPL, L1CAM, and CPLX1 are associated with the degeneration of RGCs. Lam et al. [90] conducted differential gel electrophoresis analysis on the superior, temporal, inferior, and nasal quadrants of rat optic nerve transection. The results revealed 24 differentially expressed proteins between the different quadrants. In summary, proteomic analysis on the ON model retina showed differentially expressed proteins according to the differences in modeling time, proteomic technology, and sampling sites. The signaling pathways acted on by these differentially expressed proteins are relatively few and different.

### 3.3. Application of Proteomics in Retinal Ischemia/Reperfusion Injury Research

Retinal blood vessel blockage, deformation, and prolonged poor blood flow can each cause damage to the optic nerve. At least 70 proteins are known to play a key role in optic nerve ischemic diseases. Tian et al. [91] performed proteomic analysis on the retina of optic nerve ischemic mice and found 234 differentially expressed proteins. Among these proteins, the main cellular changes they represented were related to metabolism, followed by cell transcription. The study also found that when the optic nerve was deficient after blood injury, there was inhibition of the mTOR signaling pathway and synapse-related proteins were significantly downregulated, which again confirmed that optic neuron function gradually weakened after optic nerve injury and optic neuron function was aggravated by visual nerve injury. Proteomic analysis of the retina and optic nerve in N-methyl-d-aspartic acid-induced [92] excitotoxic ophthalmopathy in rats revealed 3532 proteins in the retina and 2593 in the optic nerve. Differential protein analysis found that these proteins were associated with ferroptosis and autophagy. To further understand the protective mechanism of hydrogen sulfide (a commonly used neurotransmitter drug) on the optic nerve, Liu [68] established a rat optic nerve ischemia model and treated it with the chemical. Component analysis revealed a total of 1115 proteins, 18 of which were associated with hydrogen sulfide reduction/protection of the optic nerve from damage. Bioinformatic analysis of these differentially expressed proteins revealed that they were associated with mitochondrial dysfunction, iron homeostasis, and vasodilation activation. Zhao et al. [96] found 131 differentially expressed proteins in the rat retinal ischemia-reperfusion model, of which 24 proteins were related to histone translation modification. The authors believed that retinal ischemic injury and histone phosphorylation were associated. Vähätupa [97] used transgenic mice to establish a model of hypoxia-ischemia-induced retinopathy, used SWATH-MS technology to analyze the hypoxia-ischemia retinopathy mice, and correlated the proteomic results with human diabetic retinopathy. The results of patients with retinopathy of prematurity and retinal vein occlusion were compared and analyzed, and it was found that crystallin was one of the most abnormally secreted proteins in the early stage of retinal hypoxia-ischemia disease. The AR–Ras axis and filamin were significantly upregulated in the retina. These target proteins are known to be related to retinal neovascularization and vascular permeability.

Due to the difficulty in obtaining retina and optic nerve tissue in clinical practice, most experiments substitute this need by using animal models. Due to differences between animal species, retinal tissue preservation conditions, proteomic analysis methods, screening conditions, and experimental purposes, the same disease model study showed different results, including the number of differentially expressed proteins, the types of differentially expressed proteins, and the main action pathways, depending on these variables. Through analysis, it was found that most of the differentially expressed protein action pathways were enriched in the processes of neuronal damage, RGC degeneration and apoptosis, mitochondrial dysfunction, and carbon metabolism, among others.

### 3.4. Pathogenesis of Optic Nerve Injury Diseases

Diabetic retinopathy is a major microvascular complication of diabetes that can be divided into nonproliferative DR and proliferative DR in clinical practice. In diabetic retinopathy, the duration and degree of hyperglycemia maintenance in diabetic patients are closely related to the occurrence and progression of DR [98]. Retinal glial cells play an important role in maintaining the structural integrity of the retina and normal physiological functions [99]. After the body’s blood sugar rises, the microglia are activated, and the secretion of the inflammatory factors TNF-α and IL-6 in the cells increase, thereby promoting the secretion of VEGF. Due to chronic increases in blood sugar, the retina exists in a highly inflammatory environment for an extended period, which further leads to the dysfunction of glial cells and endothelial cells, ultimately causing the retina to be damaged by blood vessels and nerves [100,101].

When blood sugar rises, the glycolytic pathway is activated. Abnormal glucose metabolism leads to an increase in carbon metabolism, changes in glucose homeostasis in the vitreous, and changes in the vitreous environment to induce vitreous degeneration^.^ After vitreous degeneration activates the proteinase A signaling pathway, the number of endothelia in retinal blood vessels increases, the vessel wall thickens, and the diameter of blood vessels decreases, which ultimately leads to retinal hyperproteinemia, reflecting the regulation of retinal angiogenesis after diabetic retinopathy [102]. Studies have shown that when DR occurs, the “semaphorin neuron rejection signaling pathway” is inactivated, and the main regulators of the pathway (sema3A, sema3F, and sema6A) are significantly reduced. Both sema3A and sema3F can inhibit neovascularization. When the expression of sema3A in the retina is higher than that in the vitreous, sema3A induces retinal neovascularization to grow toward the vitreous, and the location of sema3A determines the growth direction of the neovascularization. The expression of sema3A in the vitreous is downregulated in DR, and sema3A can usually be injected into the lens to prevent neovascularization in the lens. Sema6A inhibits the formation of endothelial cells. Consequently, when the expression of sema6A in the retina or vitreous decreases, the production and number of new endothelial cells increases, thereby increasing their content in the vitreous and decreasing the clarity of the vitreous [103,104] (Nina et al., 2022; Guo et al., 2019).

In most cases, external forces applied to the optic nerve are indirect, causing oedema in the orbit of the optic nerve or bone degeneration, which leads to compression of the blood vessels in the retina, thickening of the spasm wall, and reduction of the diameter of the blood vessels, all of which can temporarily cause the capillaries of the arterioles to dilate. Under the stimulation of inflammatory cytokines, the vascular permeability increases, the blood flow slows down, and local edema increases, ultimately resulting in ischemia and hypoxia [91]. There is no effective method for the treatment of optic nerve injury in clinical practice. Treatments of optic nerve injury should aim to prolong the survival time of RGC, reduce the degeneration and apoptosis of RGCs, and prevent further progression to achieve the purpose of reversing the injury.

When the retinal surface blood vessels exhibit spasms, stenosis, and other symptoms, the retinal microvessels temporarily expand, the inflammatory secretions produced by the retinal cells increase, the vascular osmotic pressure increases, and the blood flow slows down, thereby resulting in retinal ischemia [105]. Retinal ganglion cells are composed of many unsaturated lipids. Following retinal ischemia, the secretion of superoxide free radicals and arachidonic acid in the retina increases, and optic ganglion cells are attacked by superoxide free radicals, which results in the production of lipids by optic ganglion cells [106].

Many studies have shown that when the internal retinal vein is occluded, the increased secretion of VEGF in the retina induces neovascularization, thereby resulting in either macular degeneration or vitreous opacity [107]. In the early stage of retinal ischemic disease, the thickness of the retina is normal, but the thickness of the optic nerve fiber layer is increased. In the middle stage of ischemia, the optic nerve begins to atrophy, and the peripheral optic nerve fibers show clear swelling, but their thickness is still less than in the early stage. In the later stage, atrophy, thinning, and visual field changes occur [107] to signify that the optic nerve has been severely damaged and many RGCs in the retina have died. Retinal ischemic diseases may be related to hereditary diseases. The principle of retinal ischemia-reperfusion disease treatment is to dilate the existing blood vessels and inhibit the formation of new blood vessels within the fundus. The main site of action was found through proteomic analysis, and it is expected that specific targeted drugs will henceforth be developed based on specific sites. These drugs can be designed to treat the disease by delaying the process of optic nerve damage and promoting the regeneration of RGCs.

## 4. The Future of Proteomics in Optic Nerve Injury Diseases

### 4.1. Shortcomings of Proteomics in the Study of Optic Nerve Injury Diseases

Optic nerve injury underlies the occurrence of eye diseases in clinical practice, and conversely, the occurrence of eye disease can also promote the development of optic nerve injury. Proteomic technology is widely used in optic nerve injury to find diagnostic targets and effective therapeutic targets for disease. The number of detected proteins between studies varies greatly, ranging from 240 to 6000 in number, which is due to differences in the proteins of different tissue sites (aqueous humor, lens, retina, and the optic nerve), different diseases, different disease models, and differences in the proteomic detection methods employed [108,109]. Although the contents and types of proteins differ between different tissues, a measurement of 6000 protein types can essentially probe them comprehensively. Among the different proteomic detection methods, there exists a large gap in the number of known differentially expressed proteins, which may indicate that the less abundant protein components are concealed when employing current methods due to different parameter settings in the experiment or sample depletion from experimental sample preparation. Protein isolation techniques are also an important reason for limiting protein quantity detection, and the total quantity of protein separated by two-dimensional gel electrophoresis is small.

Another disadvantage of proteomics is in the analysis of differentially expressed proteins. Due to different experimental purposes, the choice of data analysis method, differences in the parameter settings of differentially expressed proteins, and the number of differentially expressed proteins in each experiment are quite different. Bioinformatic analysis of differentially expressed proteins, including pathway analysis and enrichment analysis, is an important means to uncover how differentially expressed proteins play roles to mediate disease progression and outcomes. However, large-scale bioinformatic correlation analysis of differentially expressed proteins may result in some false-positive results. Given this occurrence, the error frequency should be reduced and adjusted accordingly. The pathway activities and gene enrichment results that are consistent with the differentially expressed proteins should be selected for the bioinformatic analysis.

There is difficulty in normalizing experimental proteomics data. Due to the different requirements set forth by various journals, it is difficult for researchers to reduce data for unified management, and few researchers are willing to make their data public. At the request of some journals, researchers may upload the original data of proteomics as supplementary materials. Due to differences in experimental design methods, purposes, research methods of proteomics, and sample preparation, these data are difficult to use by other researchers. This culture exacerbates the difficulty of screening diagnostic or therapeutic targets for the same diseases.

### 4.2. Future Development

The concept of proteomics was first proposed by Australian researcher Wilkins in 1994 [110]. Proteomics explores the protein composition, expression level, modification status, relationships between proteins, and the relationships between proteins and genes in cells. Proteomics has carried out an in-depth analysis of the occurrence, development, and action pathways underpinning optic nerve injury diseases, thus having provided new possibilities for the improved diagnosis and treatment of these diseases. Researchers remain committed to finding accurate diagnostic targets and effective treatment sites for different optic nerve-injuring diseases. These aim to facilitate early detection and subsequently repair optic nerve injuries in clinical medicine.

To date, proteomics technologies have continuously been improved, while the depth and breadth of protein research have also improved. In proteomic analysis, there are marked differences between the data findings due to different experimental purposes, different experimental modeling choices, and differences in sample sources and storage conditions. For these reasons, differentially expressed proteins obtained by different researchers for the same disease are very different; hence, it is difficult to obtain unified answers to research questions. The excessive number of differentially expressed proteins obtained in research is also one of the key issues that make it difficult to truly identify disease targets or therapeutic sites. Oxidative stress injury, inflammation, and glutamate pathway activation are common mechanisms underlying optic nerve injury diseases. In future studies, researchers should compare the results of this review with those of other studies of the same type, with emphasis on identifying the differences between experiments as well as differences in the obtained results.

The field of proteomics does not yet have strict executable test samples or experimental performance testing methodology standards. This may be one major reason underlying the large discrepancies between experimental results. In future research, unified test standards should be formulated for proteomics-based methodological steps, different sample processing methods, data analysis methods, and differentially expressed protein screening settings. This would be conducive to the standardized and unified operation of proteomics to reduce the impact of erroneous factors. This kind of standardization may help push the diagnosis and treatment of optic nerve-injuring eye diseases to new heights. In addition, improvements in proteins isolation, quantification, and expression pattern measurements along with qualitative animal models will improve the outcomes of results of interest, and reliable data will be generated for required databases.

## Data Availability

No new data were generated during the preparation of this manuscript.
